# Plasticity of Listeriolysin O Pores and its Regulation by pH and Unique Histidine

**DOI:** 10.1038/srep09623

**Published:** 2015-04-08

**Authors:** Marjetka Podobnik, Marta Marchioretto, Manuela Zanetti, Andrej Bavdek, Matic Kisovec, Miša Mojca Cajnko, Lorenzo Lunelli, Mauro Dalla Serra, Gregor Anderluh

**Affiliations:** 1 Laboratory for Molecular Biology and Nanobiotechnology, National Institute of Chemistry, Hajdrihova 19, 1000 Ljubljana, Slovenia; 2 Istituto di Biofisica, Consiglio Nazionale delle Ricerche & Fondazione Bruno Kessler, via alla Cascata 56/C, 38123 Trento, Italy; 3 Department of Biology, Biotechnical Faculty, University of Ljubljana, Večna pot 111, 1000 Ljubljana, Slovenia

## Abstract

Pore formation of cellular membranes is an ancient mechanism of bacterial pathogenesis that allows efficient damaging of target cells. Several mechanisms have been described, however, relatively little is known about the assembly and properties of pores. Listeriolysin O (LLO) is a pH-regulated cholesterol-dependent cytolysin from the intracellular pathogen *Listeria monocytogenes*, which forms transmembrane β-barrel pores. Here we report that the assembly of LLO pores is rapid and efficient irrespective of pH. While pore diameters at the membrane surface are comparable at either pH 5.5 or 7.4, the distribution of pore conductances is significantly pH-dependent. This is directed by the unique residue H311, which is also important for the conformational stability of the LLO monomer and the rate of pore formation. The functional pores exhibit variations in height profiles and can reconfigure significantly by merging to other full pores or arcs. Our results indicate significant plasticity of large β-barrel pores, controlled by environmental cues like pH.

Listeriolysin (LLO) is the most important virulence agent of the food-borne bacterium *Listeria monocytogenes*^1^. *L. monocytogenes* causes listeriosis, which is a severe food-borne disease, affecting primarily children, the elderly, pregnant women and immunocompromised individuals^2^. LLO is crucial for bacterial intracellular survival, as it allows escape of listeria from the acidic phagolysosomal compartment and their subsequent replication and spread through cells^3^,^4^,^5^. LLO belongs to the family of cholesterol-dependent cytolysins (CDCs), a large family of pore-forming toxins found predominately in Gram positive bacteria^6^,^7^.

CDCs are defined by their dependence on the presence of cholesterol (CHOL) in their target membranes^7^. It has been experimentally established that CHOL concentration in membranes should exceed 40 mol%^8^,^9^,^10^ for efficient binding and subsequent pore formation by CDCs, and this is true for LLO as well^11^. After binding to CHOL-containing lipid membranes, up to 50 CDC monomers oligomerise at the plane of the membrane to generate ring structures that are not yet fully inserted into the membrane and are termed pre-pores. In the final step of pore formation, each monomer of the pre-pore contributes two β-hairpins concomitant with large conformational changes to generate a huge transmembrane β-barrel that exceeds 30 nm in diameter^7^. Sometimes the protein oligomerisation at the membrane stops before completing the full ring arrangement, leading to the formation of smaller structured aggregates, termed arcs. It has been proposed that these arcs are also able to make functional pores in membranes generating a hybrid structure composed of proteinaceous β-barrel structure on one side of the pore and the lipid membrane on the other side (reviewed in Gilbert *et al.*^12^,^13^).

Pore formation by LLO and other CDCs has been shown to trigger numerous cellular effects like endocytosis^14^, histone modification^15^, activation of kinases, induction of cytokine expression and activation of NLRP3 inflammasome^16^, release of calcium from intracellular stores^17^ and inhibition of SUMOylation^18^. Some of these effects are a result of calcium or potassium flux through pores along their concentration gradients, while for others the signaling cascades are still not clear. However, LLO has also been shown to trigger cellular effects independently of its pore-forming activity. LLO is strongly immunogenic^19^ and it spontaneously aggregates lipid rafts via oligomerisation on the membrane^20^,^21^. It has also been shown that the LLO-induced mucin exocytosis develops independently of the pore-forming activity of the toxin^22^.

Listerial toxins are unique in the CDC family in their pH dependent behavior. It has been demonstrated that pH affects the stability of LLO monomers, being higher at acidic pH around pH 5.5 and significantly lower at pH 7.4, a feature absent in other non-listerial CDCs^23^,^24^,^25^. At pH 7.4 and temperatures above 30°C, LLO monomers in solution rapidly aggregate to non-functional forms^23^,^24^,^25^. This may thus be crucial for the restriction of LLO's biological activity to the intracellular acidic phagolysosomal compartment, in contrast to other non-listerial CDCs that act primarily at the level of the plasma membrane, thus at neutral pH^25^,^26^. The main function of LLO is to enable escape of *Listeria* from the phagolysosomes into the cytoplasm by inducing vacuolar destruction^1^. It has been shown that formation of pores by LLO in phagolysosome membranes is needed for the escape of listeria^27^, however the mechanistic details of this process are not yet known. Once the phagolysosomal membrane is disrupted, bacteria can multiply in the host cell cytoplasm and subsequently spread to other cells. Inactivation of LLO by the pH of the cell cytoplasm is therefore crucial for preventing its association with cellular membranes and for maintaining the integrity of the cells. However, LLO can form pores at low concentrations also in the plasma membrane of various cells at neutral pH^14^,^28^,^29^. We have previously studied LLO pore formation by various assays at different pH values and temperatures below 30°C. We found that LLO has slightly higher hemolytic and permeabilising activity at pH 5.5 in comparison to pH 7.4^23^ at temperatures lower than 37°C and that this drop in activity may be attributed to reduced binding of the protein to lipid membranes^11^. LLO is thus able to form pores also at neutral pH values *in vitro*, as long as the temperature is low enough to prevent its aggregation prior to binding to the membrane^23^. However, once bound to the membrane at a low temperature and pH 7.4, LLO effectively forms pores after the temperature is raised to 37°C^14^. *In vivo*, the perforation of the plasma membrane may be allowed by constitutive expression of LLO by extracellularly located bacteria, where despite the non-optimal conditions for LLO activity, the fraction of LLO is still active enough to form pores into the membrane^1^. While it is now clear that LLO can make pores at pH 5.5 and 7.4, the crucial question still remains whether there is any difference in the assembly, size and properties of these pores^1^.

Here we directly addressed this question. We studied the properties of pores formed by LLO using several approaches at acidic pH of 5.5–6.0, corresponding to the environment in the phagolysosome, and at physiological pH 7.4, corresponding to the conditions at the outer leaflet of the plasma membrane. Measurements of ionic currents through pores using planar lipid membrane (PLM) approach showed significant differences between pore conductance profiles at pH 5.5 or 7.4. Atomic force microscopy (AFM) can also be used to distinguish pre-pores from pores by the reduction in oligomeric height associated with membrane insertion. AFM imaging showed no difference in pore radius between the two pH values. However, it clearly showed heterogeneity in the height profiles of functional pores at either pH, indicating high plasticity of LLO pores and that membrane insertion is a gradual process for LLO oligomers. We further showed that the observed pH dependent conductance of the pore was regulated by H311, a residue unique to LLO. Mutational analysis revealed that H311 regulates the conformational stability of LLO monomers as well as kinetics of pore formation, and that the absence of this His side chain results in pH independent conductance profiles. Finally, time lapse AFM imaging showed some pores and arcs fusing into pores of irregular shape and much bigger in size than expected and this may be relevant for listerial cellular transport during infection.

## Results

### Conductance of LLO pores is pH-dependent

**Figure 1:**
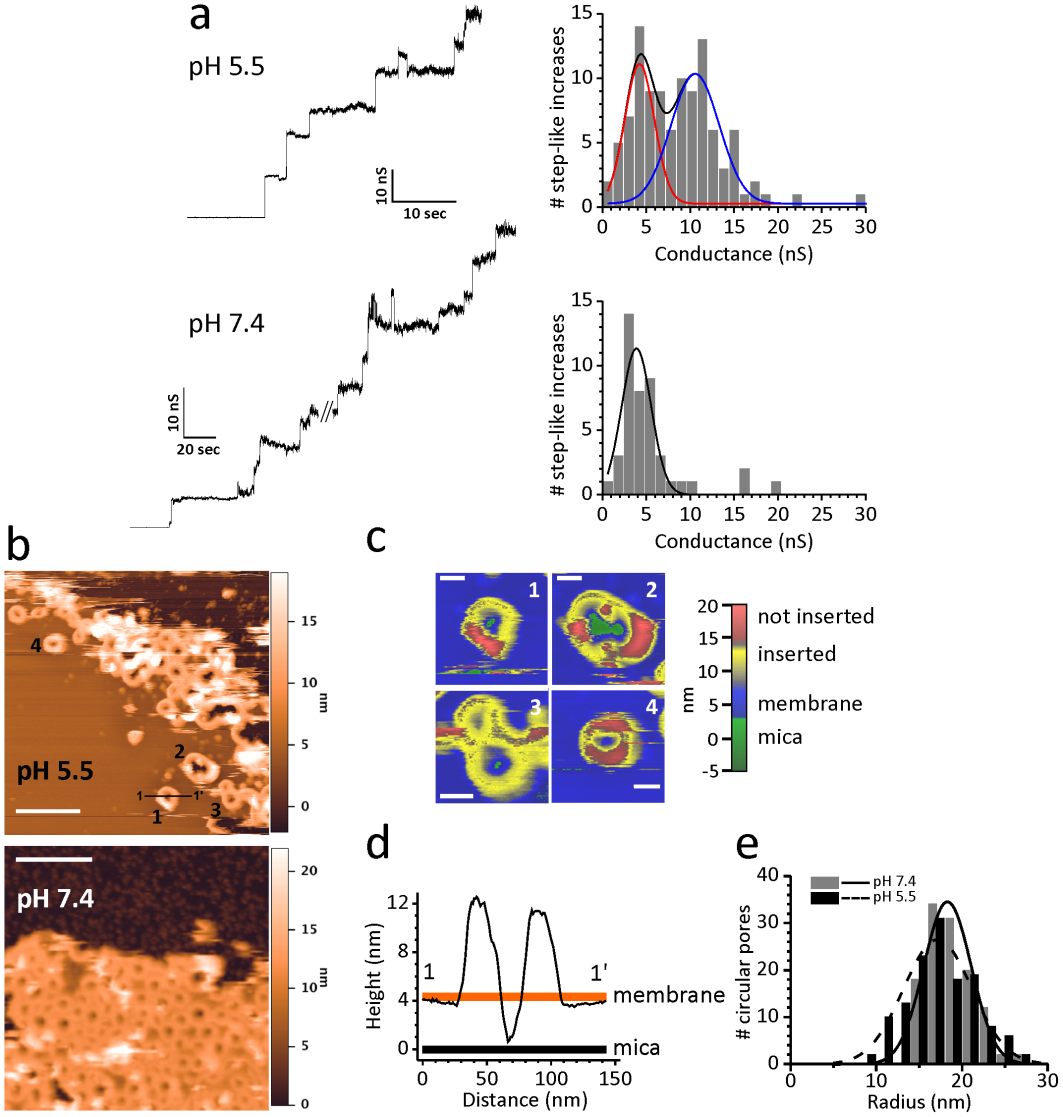
Properties of LLO pores at pH 5.5 and 7.4. (a) Conductance traces (left) of pores as measured by PLM. The protein concentration used was 5 nM or 1 nM for pH 5.5 and 7.4, respectively; the recorded current traces were obtained by applying +40 mV. Histograms of single channel conductance distribution are reported to the right of each trace. Two populations of pores that are apparent at pH 5.5 are shown by red and blue line. (b) Images of representative AFM scans of LLO oligomers formed on SLB at two designated pH values. 200 nm scale bars (white lines) are shown. Numbers (1–4) denote aggregates of various shapes not yet completely inserted and are shown enlarged in panel (c). Scale bar in (c) is 30 nm. (d) Height profiles of protein aggregates were traced along the black line shown in the AFM image in (b). (e) Distribution of radii estimated from AFM images in (b).

LLO works optimally at high CHOL concentration in lipid membranes^11^, therefore, we used membranes with 1,2-palmitoyl-*sn*-glycerophosphocholine (POPC):CHOL molar ratio 1:1 in order to study formation and properties of LLO pores. Such a membrane composition should promote the formation of full ring oligomers, as previously reported for the evolutionarily related protein perforin^30^. We used a PLM approach, which proved before to be very useful in directly studying pore-forming activity of various CDCs^31^. Examples of time courses of pore insertions for LLO at pH 5.5 and 7.4 are reported in Fig. 1A. Analysis of pore formation by LLO at pH 5.5 showed a very broad conductance distribution with two distinguished populations of pores, one of a high conductance (G_H_, 10.6 nS), including 60% of the total pores detected, and the other of a low conductance (G_L_, 4.2 nS) (Table 1). Interestingly, at pH 7.4, LLO shows significantly narrower distribution indicating only one population of pores with conductance centered at around 4 nS (Table 1), corresponding to the low conductance state observed at pH 5.5, within the experimental error.

### Size of LLO pores is pH independent

To check if differences seen in the pore conductance at the selected pH values are due to altered pore dimensions, we performed topological investigation of pores by AFM. AFM was previously successfully used for imaging pores of perfringolysin O^32^, a highly similar CDC from *Clostridium perfringens*. LLO was found to be able to bind and aggregate on supported lipid bilayers (SLB) at pH 5.5, with oligomeric rings accounting for 69% of the detected structural aggregates (Fig. 1B, Table 2). All structures protrude for at least 7 nm above the bilayer surface (Fig. 1B–D). By analogy with perfringolysin O^32^, 7 nm in height is indicative of protein that is inserted into the lipid membranes thereby forming an active pore and a height of 11.3 nm is indicative of the pre-pore structure. Interestingly, in approximately 40–50% of the LLO oligomers, arc shaped parts of oligomers with a height of 10–11 nm are clearly visible (Fig. 1C, pores 1 and 2), resulting in circular structures that could be described as only partially inserted or caught in the process of transformation from a pre-pore to pore. We succeeded to penetrate into the lumen of such aggregates with the AFM tip, down to the level of bare mica (see the height profile 1 in Fig. 1D). The absence of lipids in the center of the imaged circles strongly supports the functionality of these structured aggregates. The measured average radius of circles for LLO at pH 5.5 was 17.4 ± 0.3 nm (Fig. 1E and Table 2). We also succeeded to obtain high resolution images of LLO at pH 7.4 where 94% of structures showed the ring architecture with an average radius of 18.8 ± 0.3 nm (Fig. 1E and Table 2). Thus, AFM showed that dimensions of LLO pores are pH independent, thereby indicating that differences seen in their conductance at the two pH values tested are not due to changes in dimensions of pore structures.

### H311 as a potential regulator of pH-dependent pore conductance

**Figure 2:**
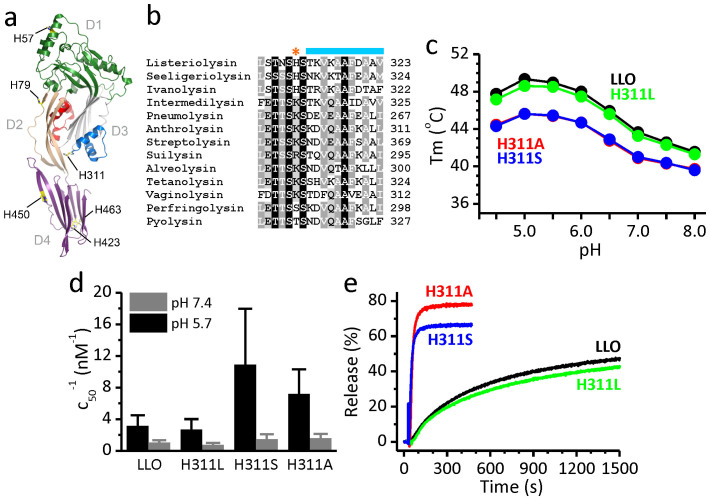
Position of the unique H311 in the structure of LLO and properties of LLO mutants. (a) Crystal structure of LLO (PDB-ID 4CDB)^33^ is shown in ribbons. Domain 1 (D1) is in green, domain 2 (D2) in light brown, domain 3 (D3) in gray and domain 4 (D4) in purple. TMH1 region is in red and TMH2 is in blue. Side chains of all histidines are shown as sticks. (b) Multiple sequence alignment of some of the CDC family members with position of H311 and part of TMH2 indicated by orange asterisk and blue line, respectively. (c) Thermal stability of LLO and mutants as described by T_m_ values measured by DSF at different pH values. All measurements were performed twice. (d) Hemolytic activity at pH 5.7 or pH 7.4. At least 4 independent measurements for each protein were performed. Hemolytic buffer and erythrocytes were either at pH 5.7 or 7.4. T = 25°C. Hemolytic activity is expressed as inverse values of protein concentration at which half maximal rate of hemolysis is achieved (c_50_). (e) Kinetics of calcein release at pH 5.5 at room temperature. Representative traces of two independent experiments are reported. Final protein concentration was 0.39 nM.

In order to understand the pH dependence of LLO pore conductance, we decided to look for residues that could act as pH-regulated switches in the LLO pore. We focused on histidine residues, since the pKa value of a free histidine is around 6.5 making it a potential candidate responsible for pH dependent features of LLO pores. LLO contains six histidine residues in its amino acid sequence (H57, H79, H311, H423, H450 and H463), spread over all four domains (Fig. 2A). We chose the residue H311 as potentially the most interesting, since it is located in the domain 3 (D3) at the beginning of the transmembrane helix (TMH) 2 region, at the interface between domains D2 and D3 (Fig. 2A)^33^. This part of the LLO structure is expected to undergo large structural changes upon unfurling of the monomer and aggregation at neutral pH in the cytosol or during the protein insertion into the membrane. These helices are suggested to transform into β-strands forming the wall of the pore channel contributed by transformed TMHs of all monomeric units of LLO building the pore^34^. Importantly, H311 is unique to LLO, found only in listerial strains, while other, non-listerial members of the CDC family contain Lys, Ser, Arg or Thr at the equivalent position (Fig. 2B). We prepared several LLO single mutants. Mutation to Ser was selected according to the presence of this residue at the equivalent positions in other CDC members. Mutation to Ala or Leu was used to convert the side chain to a small or middle-sized hydrophobic residue, respectively. We also attempted to prepare H311K, but it was extremely poorly expressed and the growth of *Escherichia coli* was inhibited in the case of the H311E mutant. Therefore, all further studies were performed with LLO and its mutants H311A, H311S and H311L, which were purified to a high degree and showed no significant difference in behavior during purification in comparison to the wild-type LLO (Supplementary Fig. 1). Their CD spectra at room temperature were comparable in shape (Supplementary Fig. 2), indicating no significant changes in the secondary structures of the proteins due to mutation.

### Mutation of H311 affects stability of LLO monomers and enhances pore formation

First we checked the impact of the mutation at position 311 on the stability and permeabilisation properties of LLO. Thermal unfolding was followed in the pH range 4.5–8.0 by differential scanning fluorimetry (DSF) (Fig. 2C), with the melting temperature (T_m_) determined from the inflection point of the melting curve at each pH as a measure of the protein stability^35^. A plot showing dependence of T_m_ on pH reveals a bell-shaped profile for all LLO variants (Fig. 2C), with the highest T_m_ value for LLO close to 50°C at the acidic pH 5.0–5.5, dropping close to 42°C at pH 7.5. This is in agreement with previous studies, which showed that LLO has generally low stability at neutral pH as a monomer in solution^23^,^24^. While the introduction of the mutation at position H311 to Ala or Ser retains the bell shaped profile of T_m_ versus pH similar to LLO, the absolute value of T_m_ for these two mutants in all the pH range is lowered by approximately 4°C in comparison to LLO, indicating a diminished stability of the mutated proteins. In contrast, the melting profile for H311L mutant shows a similar thermal stability to LLO. Additional assessment of the impact of a single mutation on the overall structural stability of monomeric protein was done with circular dichroism (CD) far-UV spectroscopy at pH 5.7 and 7.5 (Supplementary Fig. 3). Interestingly, all LLO variants showed stability across the temperature range 20–50°C at pH 5.7, while a typical melting curve was detected at pH 7.5 (Supplementary Fig. 3). LLO and the H311L mutant showed comparable melting temperatures of the secondary structure, i.e. 35°C, while the mutants H311A and H311S start to melt at a slightly lower temperature, 33°C (Supplementary Fig. 3), indicating lower stability of the secondary structure of H311A and H311S mutants in comparison to LLO and H311L. These results suggest that the absence of H311 imidazole side chain does not affect the typical pH stability of LLO, being highest at acidic conditions for all tested proteins. The drop of T_m_ in the case of mutation to the smaller side chains of Ser and Ala indicates the importance of a bulkier residue like His or Leu at position 311 for the stability of LLO monomer in solution.

The permeabilising ability of LLO and its mutants was evaluated using two assays with different lipid bilayer systems. First we measured the hemolytic activity of LLO variants. The assay was performed at pH 5.7 and 7.4 (Fig. 2D). All proteins showed higher lytic activity at pH 5.7 than at pH 7.4. Interestingly, H311A and H311S showed higher hemolytic activity than wild type LLO and H311L at both pH values. To further demonstrate that the chemical nature of the amino acid side chain at position 311 affects the pore formation, we measured calcein release from LUVs in the presence of LLO or mutants. The pore formation was again significantly faster for H311A and H311S mutants, than for LLO or H311L (Fig. 2E), in accordance with hemolytic activity described above. In summary, both permeabilising assays indicated that higher lytic activity is achieved by thermally less stable LLO variants with Ser or Ala at position 311 in comparison to the ones with bulkier Leu or original His side chain. We also tested the toxicity of all LLO variants on cultured human, undifferentiated intestinal epithelial cell line Caco-2. A survival rate of Caco-2 cells was measured upon addition of LLO variants (Supplementary Fig. 4) at pH 6.0 and 7.4. The Caco-2 cell viability was affected to the same extent for all LLO variants at either pH value, indicating that the pores formed by different proteins, although with potentially different kinetics and conductance properties, were still sufficient to disturb the integrity of cells to the same extent at the chosen concentration of the added toxin.

### H311 is crucial for the specific pH dependent conductance profile of LLO pores but does not affect the pore size

**Figure 3:**
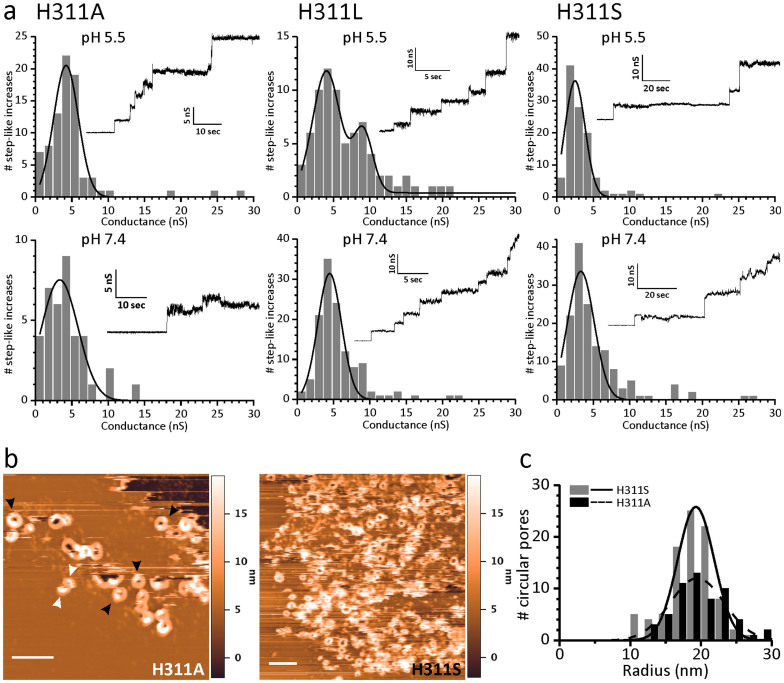
Pore properties of LLO mutants at pH 5.5 and 7.4. (a) Conductance of pores as measured by PLM. The protein concentration was 5 nM or 1 nM at pH 5.5 and 7.4, respectively. Histograms of single channel conductance distribution are reported together (inset) with the recorded current traces that were obtained applying +40 mV. (b) AFM images of pores formed by LLO mutants on SLB at pH 5.5. 200 nm scale bars (white lines) are shown. Black arrowheads denote parts of rings not yet completely inserted, while white arrowheads denote arcs that also are not inserted. (c) Distribution of the radii estimated from AFM images shown in (b).

The experiments above showed that the presence of a small side chain at position 311 significantly affected stability of monomeric LLO as well as the kinetics of pore formation. We used PLM approach in order to test whether mutations at position 311 also affect the pH dependent conductance of LLO pores. All mutants were able to induce well resolved step like-increases in conductance, both at pH 5.5 and 7.4 (Fig. 3A, Table 1). At pH 5.5, H311L mutant exhibited a profile similar to the wild type LLO with two conductance states of 9.0 and 4.0 nS, for G_H_ and G_L_, respectively. However, H311L pores belonging to a high conductance population were less conductive than the corresponding ones of the wild type, and they represented only 27% of the total pore population (Fig. 3A, Table 1). H311A and H311S showed only one population of pores, corresponding to the low conductance state of the wild type LLO (Fig. 3A, Table 1). At pH 7.4 all mutants showed a broad conductance distribution centered at around 4 nS, similar to the wild type LLO (Fig. 3A, Table 1). Therefore, the replacement of His side chain at position 311 reduces the conductance of LLO pores at pH 5.5 to only one main population with low conductance values, and this feature remains unchanged at pH 7.4.

Pores formed by mutants were also imaged by AFM at pH 5.5 (Fig. 3B), however, due to the high SLB destabilizing activity of the mutants at pH 7.4, the AFM imaging could not be performed at this pH. H311A and H311S mutants bound and aggregated on SLB at pH 5.5, while H311L binding strongly destabilized SLB structure and did not allow any further structural characterization by AFM. H311A and H311S formed pores of similar shape and size as the wild type LLO (Table 2). The measured radii of the full circular aggregates were 19.7 ± 0.5 and 18.8 ± 0.3 for H311A and H311S, respectively (Fig. 3C). As already observed in the case of the wild type LLO, the mutants formed many full rings that were only partially inserted (black arrowheads in Fig. 3B; see also arcs that were not reduced in height to the inserted states, labelled by white arrowheads). Also in this case, we were able to penetrate with the tip into the lumen of the pore to the level of the mica, showing that mutant pores were no different in their architecture from those of the wild type LLO.

### Plasticity of pore assembly by LLO

**Figure 4:**
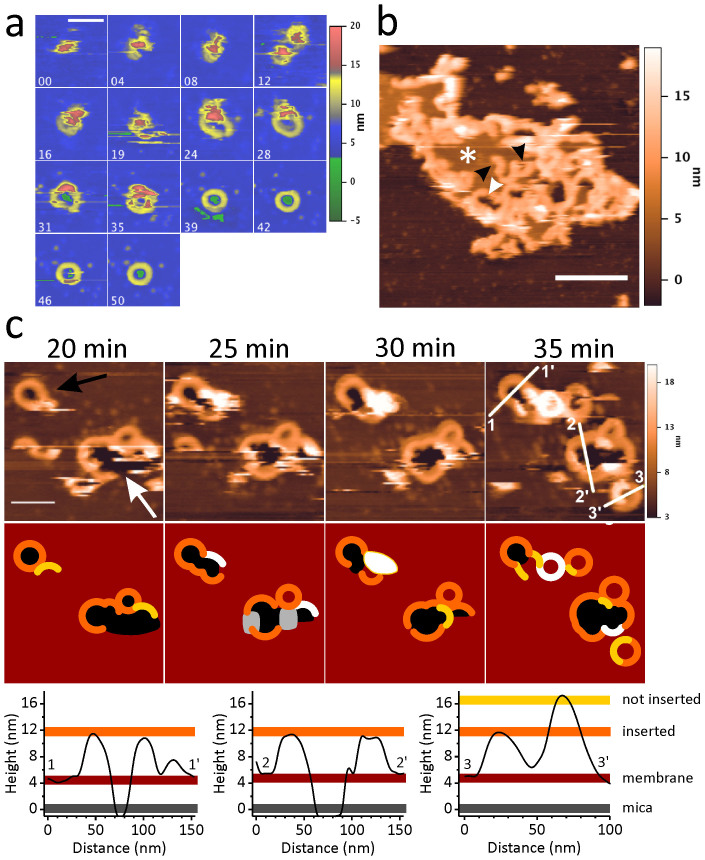
Dynamics of LLO pore formation as imaged by AFM. (a) Time course of pore formation by the wild type LLO on SLB at pH 5.5. Numbers at the upper left side of each image show the time in minutes. The color code on the height scale is the same as in Fig. 1C; scale bar length corresponds to 100 nm. (b) Scan of the wild type LLO oligomers on SLB at pH 5.5. White asterisk marks a lipid bilayer patch. Black arrows indicate arc-like structures. A large porous structure formed by arcs facing each other is marked by white arrow. Scale bar length corresponds to 500 nm. (c) Time evolution of oligomers formed by the H311A mutant on SLB at pH 5.5. Upper part: Four AFM scans, acquired with a 5 minutes time interval, show the growth and fusion of new structures with an already formed ring (black arrow) and the closure of a large structure, mainly consisting of or being contoured with joined arcs (white arrow). The time indicated above the panels was counted after injection of the protein across SLB. Below: sketch reconstruction of AFM scans in the upper panel to clearly depict the shape and height of various protein aggregates. Height profiles of protein aggregates along the white lines shown in the AFM image in (c) after 35 min are shown below the image.

In the membrane model system employed in our experiments, the wild type LLO showed a tendency to form complete circular pores. We succeeded to catch the assembly of isolated pores from an initial aggregate that slowly grew into an ordered structure (Fig. 4A and Supplementary Movie for representative images). During this process, parts of the aggregate started to transform into a lower state (yellow in Fig. 4A), ending into a uniform, inserted ring. We were also able to evidence a perturbation of the lipid membrane inside the ring that was not yet completed (shown in green in Fig. 4A). Sometimes arcs could also be found on a patch of the lipid membrane, as shown in Fig. 4B (denoted by black arrows). Some of the arcs showed a certain degree of flexibility, as they coalesced to form irregular or double pores (Fig. 4B, denoted by white arrows). The height profile of the protein and the absence of the lipid bilayer from the middle of the aggregated structures (black) indicated that arcs were fully inserted and thus functional. We also collected four consecutive AFM images that revealed temporal rearrangements of H311A oligomers adsorbed into SLB (Fig. 4C). From this sequence it seems that the insertion of a new oligomer is facilitated in close proximity of a larger already inserted ring-shaped oligomeric structure, as a seed, which may then open and fuse with the new growing structure, as has also been recently hypothesized^33^. These AFM images further reveal that LLO pores are not rigid structures but rather flexible with ability to reorganize at the surface of the lipid membrane and that partially formed rings are able to insert into the membrane to form functional pores as well (Fig. 4C).

## Discussion

LLO is unique among members of the CDC family for being sensitive to the pH of its environment^23^,^24^. High stability of the LLO monomer in solution at acidic conditions and its significant drop at neutral pH at temperatures above 30°C, concomitant with aggregation and thus deactivation of pore forming activity^23^,^24^, is most possibly the key feature of LLO that enables the intracellular activity of *L. monocytogenes*. pH dependent behavior of LLO has been mainly attributed to the presence of an acidic triad in domain D3, designated also as the pH sensor^24^ that initiates the denaturation of LLO monomer at neutral pH by domain D3 destabilization. However, there is growing evidence of LLO activity before bacterial entry, revealing that pores can actually be formed by LLO efficiently also at the extracellular site of the plasma membrane at non-optimal conditions of neutral pH and 37°C^14^,^15^,^18^,^29^,^36^. It is thus vital to study structural properties of LLO monomer as well as its pore formation activity under various conditions in detail in order to understand the mechanism and role of LLO in different cellular contexts^1^. Furthermore, LLO was shown to produce pores of significantly different size, which could lead to different responses during infection^27^,^36^,^37^. This opens further important questions, whether there is a difference in pore size between the acidic and neutral pH, and how the environment including constituents of different cellular membranes affects the pore formation, i.e. whether the pores formed in the plasma membrane are similar in physical and chemical properties to the ones formed in the phagolysosomal membrane.

Our study shows for the first time that at acidic pH and on cholesterol-enriched membranes, two main populations of pores are formed by LLO, the more populated one showing almost 3-times higher ionic conductance than the other population, while at physiological pH, only one population was found, corresponding to the low conductance state found in acidic conditions. Importantly, we show that these dramatic differences between the pore characteristics at two distinct pH values were not caused by changes in outer pore shape or diameter, suggesting that some other internal physical or chemical property of the pore lumen might tune this pH dependent feature of LLO pores. Assuming that H311 is the sole responsible of such a dramatic change in conductance in response to pH and taking into account the dimensions and pKa of His side chain, we could hypothesize that the environment around H311 could affect the conductance of the LLO pore lumen. For example, at acidic pH, most of the His side chains are expected to be positively charged, while being mostly neutral at the neutral pH. Furthermore, due to the existence of the two possible charge states, H311 side chain could be differently engaged at different pH values, either making interactions with the pore wall or pointing into the pore lumen. Consequently that would influence the ion flux, being higher at acidic conditions in comparison to the neutral pH. Thus, large β-barrel pores may not just be considered as simple static perpendicular transmembrane hollow cylinders, but have a more complex and dynamic internal structure, which does not affect the external appearance, detected by structural methods like electron microscopy (EM) or AFM. Similar features were observed for another CDC member, pneumolysin, and its mutant W433F^38^,^39^, which shared pores of the same shape and diameter as observed by EM, but of different activities in terms of conductance, polymer effect on channel activity or sensitivity to ions.

Besides the pH regulation of LLO pore conductance we also noticed a significant plasticity in the height of pores and arcs, however, this feature proved to be pH independent on our membrane model system. The measurements of heights of LLO aggregates on the membrane by AFM suggested that while they seemed functional, i.e. perforating the membrane, they were only partially inserted (Figs 1B,C and 4). Furthermore, while most of the LLO aggregates formed ring structures, we noted an additional LLO tendency to form growing arc structures, which aggregated and fused, thereby forming irregular or double pores (Fig. 4B, C). Also in this case, the height profile of protein particles and the absence of lipid bilayer in the middle of aggregated structures indicated fully inserted and functional arcs and other pores of irregular shape. Moreover, time-resolved AFM imaging of pore formation revealed a sequence of events showing that formation of a new ring from an arc was facilitated by the close proximity of already formed rings, opening and fusing with new growing structures, with rings as well as arcs being able to efficiently protrude into the membrane to form functional pores (Fig. 4A, C). Therefore, we show evidence that LLO pore formation is a very dynamic process with oligomeric clusters of LLO (pores, arcs, double pores and even larger pores of irregular shape) as flexible structures actively reorganizing at the surface of the lipid membrane. This dynamics is a newly recognized feature of the pore assembly, which was recently reported also in the case of lysenin, a β-barrel pore forming toxin from the earth worm^40^. Using a real time visualization of the pore formation by high speed AFM imaging, highly dynamic randomly forming individual clusters were observed initially, including lysenin oligomers of irregular size and shape, diffusing, dissociating and associating along the membrane. As the number of the round-shaped clusters increased with time, randomly forming clusters gradually diminished. In addition to the reorganization of the clusters along the membrane, the changes in the cluster height were also observed, revealing two distinct populations, the taller one possibly representing the pre-pore like formation and the lower one representing the inserted lysenin^40^. Therefore, lysenin first forms individual clusters of various size, which diffuse along the surface of the membrane to arrange into highly ordered structures. The presence of different classes of oligomers was also observed by AFM for another β-barrel forming toxin, γ-hemolysin, also suggesting to be considered as a sign of the physiological dynamics towards the final structure formation in which the complexes with a lower number of subunits are intermediate states on the way to the fully active octameric pore arrangement^41^.

In addition to the previously recognized pH-dependent stability of LLO monomer attributed to the acidic triad in the D3 domain^24^, we show here that the pH dependent plasticity of the LLO pore is due to the presence of His side chain at position 311. In fact, this residue plays a triple role in structural and functional properties of LLO by affecting (i) structural stability of the soluble monomer in the broad pH range, (ii) kinetics of the pore formation process, and (iii) pH dependent pore conductance. Increased permeabilising activity of Ala and Ser mutants indicate that the rate of pore formation is in part regulated by stability of the domain D2/D3 interface in LLO monomer, i.e. the mutants of lower stability H311A and H311S tend to oligomerise faster into presumably energetically more favorable pore than the more stable wild-type protein or H311L mutant. According to the model of a CDC pore^42^ and the position of H311 just before the starting of TMH2 region^33^, it can be expected that the H311 position in the oligomeric pore would be at the upper rim of the lipid bilayer. Thus, its side chain could be pointing either into the pore lumen or into the aqueous region between unfolded D3 and reconfigured positions of D2 and D4 domains^42^. Our experiments show that the chemical nature of the imidazole side chain of histidine seems to modulate the pore conductance in pH dependent manner, indicating that H311 side chain points into the lumen of the pore. This effect is not detected for residues that do not contain any ionisable side chain, like Ala and Ser; even Leu mutant was more similar in conductance profile to Ala and Ser mutants than to the wild-type LLO, considering that its higher conductance state is less populated and shows a smaller conductance (Table 1). pH dependent conductance profiles could be thus partially attributed to the positive charge of His side chain at pH 5.5, which becomes neutralized at pH 7.4. The presence of a titratable bulky His side chain at this position may be able to modulate the effective lumen dimension according to pH. Koster *et al*.^33^ estimated pKa of H311 to be 5.6 and therefore at pH used in our electrophysiological experiments, i.e. 5.5, only half of the His should be positively charged, and this could explain the presence of two conductance states for LLO at the acidic pH (Fig. 1A). This pH dependence of LLO pores supports the idea of the formation of different pores at the plasma or vacuolar membrane, as also suggested by Hamon *et al.* 2012^1^, with their properties mediated by the pH of the respective environments. According to our results, pH affects the effective functional size of LLO pores, but not their actual physical architecture at the membrane surface, which may result in differences in the flux and selection of either smaller components like ions as well as bigger molecules including proteins under different environmental conditions.

Diverse pore structures were reported also for the structurally related human perforin, a protein belonging to the MACPF/CDC protein superfamily^12^. We have shown recently that human perforin pores exhibit a broad range of conductances, from 0.15 to 21 nS^30^, which are in this case modulated by the membrane lipid composition. Less ordered membranes (where DOPC is present as a component) allowed prompt insertion of perforin small oligomers with lower conductance and higher noise level in ionic current, while more ordered acyl chains (where POPC is the main component, as used in this study) prevented insertion of oligomers until the full ring-shaped pre-pore was formed. Similarly to perforin, we recently reported a broad distribution of LLO pore conductances in DOPC containing membranes with average values of 1.4 pS or 2.2 pS at pH 5.5 or 7.5, respectively^23^. These values were much smaller than those reported here for POPC:CHOL membranes, which favor the full ring formation, as clearly seen from AFM images and less noisy conductances with values higher than 4 nS. The small conductances measured in less ordered DOPC containing membranes are supportive of a massive presence of incomplete, but functional arcs^23^. In this paper we reported evidences that the high and low conductance state in POPC:CHOL membranes both derive from ring shaped pores. pH dependence of LLO pore conductance and effects of lipid composition of target membranes may be relevant for the regulated formation of pores by LLO in different compartments *in vivo*. Pores formed at the plasma membrane with a reduced ion flow due to the neutral pH and maybe also different selectivity might prevent the irreversible damage of the membrane, while inhibiting the host's immune response^15^. On the other hand the presence of more conductive pores may be necessary for the intracellular invasion through disruption of the phagolysosome and release of the bacterium into the cytosol where it can replicate and continue its life cycle. The underlying feature, however, is that large β-barrel transmembrane pores, such as large LLO pores studied here, are highly dynamic structures that are responding readily to environmental conditions such as pH or lipid membrane composition.

The evidence of the highly dynamic mechanism of LLO pore formation shown here with arcs fusing with complete pores as nuclei to either form new regular round pores, as the main population of pores, or optionally, much larger porous aggregates with capability to bud into regular pores as well, may support the temporal dynamics at the phagolysosomal membrane suggested by Shaughnessy *et al.*^27^. Namely, these authors suggested that in the first minutes after infection of macrophage vacuoles, *L. monocytogenes* secretes LLO, which creates small perforations in vacuolar membranes that allow exchange of calcium, protons and small molecules. Subsequent expansion creates larger perforations that allow exchange of macromolecules and the eventual delivery of the bacterium into the macrophage cytosol. Such larger perforations could correspond to fused pores or porous aggregates as shown in Fig. 4. Furthermore, a significantly larger portion of regular round pores at the neutral pH (Table 2) may indicate that the process is less dynamic at this condition. Other time resolved methods than AFM may enable complementary and more detailed studies of LLO pore formation in membranes with various lipid compositions at acidic and neutral pH. Based on the results of this study we expect that our observations of pH-dependent plasticity of LLO pores *in vitro* may translate into the mechanism of pore formation *in vivo*.

## Methods

### Materials

All materials were from Sigma, unless specified otherwise. Oligonucleotides were purchased from MWG (Germany). 1,2- palmitoyl -*sn*-glycero-3-phosphocholine (POPC) and cholesterol (CHOL) were from Avanti Polar Lipids.

### Cloning and Protein Preparation

A construct to express LLO (residues 25–529) was prepared in pPROEX-HTb vector (pPROEX-HTb-LLO) by using standard molecular biology approaches and LLO gene as a template^23^. LLO H311 mutants, H311A, H311K, H311E, H311S and H311L were prepared by site-directed mutagenesis of pPROEX-HTb-LLO, carried out essentially as described in Ref. 43. All constructs included a Tobacco Etch Virus (TEV) protease cleavage site inserted between N-terminally placed hexa-histidine tag and the beginning of the target gene. All constructs were confirmed by sequencing (GATC Biotech, Germany).

Recombinant proteins were expressed in the *E. coli* BL21(DE3)pLysS strain on induction using 500 μM isopropyl-beta-thio galactopyranoside for 20 h at 20°C. Cells were lysed by sonication in a buffer containing 50 mM NaH_2_PO_4_ (pH 6.5), 5 mM 2-β-mercaptoethanol, 250 mM NaCl, 10% glycerol, and 2 mM phenylmethanesulfonylfluoride, followed by centrifugation at 17,000 *g*. The supernatant was interacted with Ni-NTA (Qiagen) beads and the matrix was washed with the buffer containing 50 mM NaH_2_PO_4_ (pH 6.5), 300 mM NaCl, 25 mM imidazole and 5% glycerol, and the histidine-tagged protein was eluted with similar buffer containing 300 mM imidazole. The eluted protein was dialyzed over night at 4°C against 20 mM Tris-HCl pH 7.0, 200 mM NaCl and 5% glycerol in the presence of TEV protease in order to remove the hexa-histidine tag. The remnant histidine-tagged protein and TEV protease were removed by passing the sample through Ni-NTA column using 50 mM Tris-HCl pH 7.0, 250 mM NaCl, 10 mM imidazole and 5% glycerol, while the non-bound fraction including the purified LLO was collected and concentrated using AmiconUltra (Millipore) concentrators and the buffer was simultaneously exchanged to 20 mM Mes pH 5.7, 150 mM NaCl, 5% glycerol and 2 mM DTT. The purified proteins were stored in aliquots at −80°C until further use.

### Differential Scanning Fluorimetry (DSF)

DSF was used to determine thermal stability of proteins in the pH range 4.5–8.0 (in 0.5 unit intervals) using real-time-PCR (Roché LightCycler® 480) and 96-well system (LightCycler® 480 Multiwell Plates, 96-well, white) and temperature range 20–85°C, with a gradient 1.8°C/min. Buffers used were 20 mM Na-Acetate for pH range 4.5–5.5, and 20 mM Na-Phosphate for pH range 6.0–8.0. In all buffers, the concentration of NaCl was 150 mM. Thermal stability at various concentrations of salt was measured at pH 5.7 (20 mM Na-Acetate) and 7.5 (20 mM Na-Phosphate). NaCl concentrations used were 0, 10, 50, 150, 200, 300 or 500 mM. The final protein concentration in the well was 0.1 mg/ml (1.8 μM). Fluorescent dye Sypro® Orange (Invitrogen) was diluted in ultra pure water before the experiment (5000 × stock solution) and 2 × dye solution was used as a final concentration in the well. Data were analyzed using Excel script for the analysis of protein unfolding data acquired by DSF^35^,^44^. The transition midpoint between native and denatured protein population, i.e. the melting temperature of the protein (T_m_), was calculated using Boltzmann function in Origin 8.1 (OriginLab Corporation). All measurements were performed twice.

### Hemolytic activity assay

Hemolytic activity was measured in terms of attenuance on bovine erythrocytes at 25°C using microplate reader (BioTek, USA), essentially as described in Ref. 45. LLO and mutants were added into the first well to the erythrocyte buffer (20 mM Mes, pH 5.7 or 20 mM Tris-HCl pH 7.4, and 140 mM NaCl), to a final concentration of 10 nM, and then serially diluted two-fold. 100 μL of washed bovine erythrocytes with A_630_ = 1 in erythrocyte buffer were added to 100 μL of the protein, and hemolysis was monitored by measuring attenuance at 630 nm for 20 min at 25°C. We found no significant difference in hemolytic activity of LLO in the pH range pH 5.5–6.0 and, therefore, all further experiments with regard to low pH were done in this pH range.

### Calcein Release

Large Unilamellar Vesicles (LUVs) were prepared by extrusion through 100 nm pore filters and loaded with calcein at the self-quenching concentration (approximately 80 mM)^46^. LLO lytic activity was tested following the fluorescent calcein signal at 538 nm. The excitation wavelength was set to 495 nm. LLO and mutants were tested using LUVs composed of POPC:CHOL 1:1 (mol:mol). Calcein loaded LUVs were exposed to two different LLO concentrations (0.4 nM and 3.1 nM) in the buffer 10 mM NaH_2_PO_4_, 150 mM NaCl, 1 mM EDTA, pH 5.5. The experiments were performed on a spectrofluorimeter (Fluoromax 4, Horiba Jobin-Yvon) and the percentage and the kinetics of the lytic activity were estimated on the basis of the total fluorescence achieved by addition of detergent Triton-X100 to a final concentration of 1 mM.

### Planar Lipid Membranes

Solvent-free PLM of POPC:CHOL 1:1 (mol:mol) were prepared and measurements performed as described in Ref. 47. Briefly, two chambers, separated by a teflon septum, were filled with 2 ml of buffer and a planar membrane was formed on the septum hole. Lipids were added from 5 mg/ml pentane solution. The buffers used were 10 mM MES, 100 mM KCl, pH 5.5 and 20 mM Hepes, 100 mM KCl, pH 7.4. Proteins were added at 1–5 nM concentration in the *cis* chamber (where the potential is applied, while *trans* chamber was grounded). Recordings of the current were performed by Axopatch 200 (Axon Instruments) at the constant applied voltage of +40 mV and data were acquired by a PC equipped with a DigiData 1200 A/D converter (Axon Instruments). The current was filtered at 0.5 kHz and acquired at 2 kHz using Axoscope 8 software (Axon Instruments). The measurements were carried out at room temperature. Solutions on each side of the membrane were stirred by miniature magnetic stir bars. The conductance (G) was determined as follows:
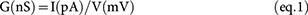
where I is the ionic current through the membrane, and V is the applied transmembrane potential.

### Atomic force microscopy (AFM)

Supported lipid bilayers (SLB) were prepared by liposome fusion technique, according to the procedure described in Ref. 48. Briefly, liposome preparation (POPC:CHOL 1:1 (mol:mol)) was incubated on a freshly cleaved mica surface for one hour to obtain the formation of a homogeneous supported lipid bilayer. The excess lipids in suspension were removed by a washing step. In some cases, to avoid any membrane perturbation due to the washing steps, successive apposition of two POPC:CHOL monolayers on the freshly cleaved mica was performed (similarly to Ref. 49).

The proteins were incubated with the SLB for 45 min at the final concentration of 6 nM. Unbound protein was gently removed by perfusing 10 mM MES pH 5.5, 150 mM KCl buffer with an automatic perfusion system (sp260p syringe pump, WPI). In experiments at pH 7.4 the perfusing buffer was 20 mM HEPES with 150 mM KCl. Time-lapse AFM experiments were performed adding the protein directly to the SLB mounted in the AFM chamber and acquiring successive scans of the same region with a ~5 minutes interval. Image acquisition was performed using the droplet tip holder of the AFM instrument (Cypher, Asylum Research). Images were acquired in AC mode using Olympus BL-AC40TS cantilevers (nominal force constant of 0.9 N/m) with the Asylum provided routines running in Igor Pro 6.12A (WaveMetrics, USA). The acquired data were visualized and analyzed with Gwyddion 2.31 (http://gwyddion.net/) and ImageJ^50^.

## Supplementary Material

Supplementary Information Supplementary Information

Supplementary Information Supplementary Movie

## Figures and Tables

**Table 1:** Pore conductances (G) measured by PLM

Protein	pH	G^1^ (nS) ± FWHM	#^3^ pores
LLO at G_H_	5.5	10.6 ± 5.5 (60%)^2^	107
LLO at G_L_	5.5	4.2 ± 3.3 (40%)^2^	
H311L at G_H_	5.5	9.0 ± 2.6 (27%)^2^	74
H311L at G_L_	5.5	4.0 ± 3.7 (73%)^2^	
H311A	5.5	4.2 ± 1.6	82
H311S	5.5	2.5 ± 2.6	109
LLO	7.4	3.9 ± 3.3	45
H311L	7.4	4.5 ± 3.2	126
H311A	7.4	3.3 ± 5.0	38
H311S	7.4	3.3 ± 3.6	152

^1^conductance and full width at half maximum (FWHM) from Gaussian fit; G_H_ and G_L_ high and low G values, respectively.

^2^percentage of the total number of pores belonging to the reported conductance distribution from the Gaussian peak area.

^3^number of pores.

**Table 2:** Properties of oligomeric aggregates observed by AFM

Protein	pH	r^1^ (nm) ± SEM	#^2^ structures	% rings
LLO	5.5	17.4 ± 0.3	132	69
LLO	7.4	18.8 ± 0.3	119	94
H311A	5.5	19.7 ± 0.5	57	79
H311S	5.5	18.8 ± 0.3	90	59

^1^pore (ring) radius.

^2^number of structures observed.
