# Corrigendum: Plasticity of Listeriolysin O Pores and its Regulation by pH and Unique Histidine

**DOI:** 10.1038/srep15690

**Published:** 2015-11-06

**Authors:** Marjetka Podobnik, Marta Marchioretto, Manuela Zanetti, Andrej Bavdek, Matic Kisovec, Miša Mojca Cajnko, Lorenzo Lunelli, Mauro Dalla Serra, Gregor Anderluh

Scientific Reports 5: Article number: 9623; 10.1038/srep09623published online: 04082015; updated: 11062015

The original version of this Article contained a typographical error in the title of the paper, where the word “Listeriolysin” was incorrectly given as “Lysteriolysin”.

In addition, there was a typographical error in the legend of Figure 4:

“(a) Time course of pore formation by the wild type LLO on SLB at pH 5.5. Numbers at the upper left side of each image show the time in seconds”.

now reads:

“(a) Time course of pore formation by the wild type LLO on SLB at pH 5.5. Numbers at the upper left side of each image show the time in minutes”.

These errors have now been corrected in the PDF and HTML versions of the Article.

