# Supplementary material for: Plasticity of Listeriolysin O Pores and its Regulation by pH and Unique Histidine
Source: Sci Rep. 2015 Apr 8;5:9623. doi: 10.1038/srep09623 (PMC5381700; doi:10.1038/srep09623)
Supplement: Supplementary Information [file srep09623-s1.pdf]

Plasticity of Lysteriolysin O Pores and its Regulation by pH and Unique Histidine

Marjetka Podobnik, Marta Marchioretto, Manuela Zanetti, Andrej Bavdek, Matic Kisovec, Miša Mojca Cajnko, Lorenzo Lunelli, Mauro Dalla Serra, Gregor Anderluh

SUPPLEMENTARY INFORMATION

Supplementary Movie: Formation of pore by LLO.

Supplementary Methods

Circular Dichroism (CD) Spectroscopy

CD spectra were collected on Chirascan CD Spectrometer (Applied Photophysics, UK). Proteins were diluted in the respective buffer to a final concentration of 0.2 mg/ml. Spectra were taken at pH 5.7 (20 mM MES, 10 mM NaCl) and 7.5 (20 mM Tris-HCl, 10 mM NaCl), as well as at different temperatures with discrete temperature steps.

Viability of Caco-2 cells

Caco-2 cells were cultured on 96-well microtiter plates for 1 week in DMEM D5921 (pH 7.4) (Dulbecco's modified eagle medium) with 1 % glutamine (PAA – The cell culture company), 1 % Antibiotic-Antimycotic mixture (GIBCO) and 10 % FBS (fetal bovine serum). For experiments at pH 6.0, DMEM D5523 was used, upon addition of 1 % of the mixture 25 mM MES pH 6.0 and 0.23 g/ml NaCl. Prior to treatment, cells were washed with serum free medium of pH 7.4 or 6.0. Cells were then treated with different LLO concentrations and incubated for 3 hours at 37 °C in the buffer with respective pH. After 3-hour incubation cells were washed in the test medium described above (pH 7.4) and then incubated overnight at 37 °C. MTT cell viability reagent was then added to the cells followed by 2 hour incubation at 37 °C. The formed formazan crystals were then dissolved in DMSO and absorbance measured at 570 nm. Experiments were done twice for each pH and each time in two parallels.

Supplementary Figure 1

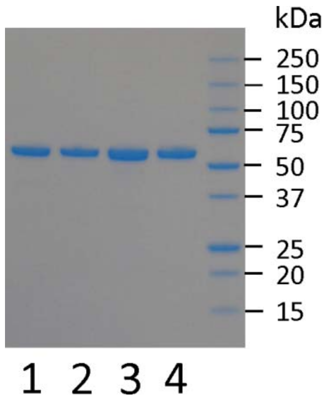

Supplementary Figure 1. SDS-PAGE gel of LLO and its mutants

SDS-PAGE of purified proteins used in this study was stained with SimplyBlue^{TM} SafeStain (Life Technologies, USA). 1, wild-type LLO; 2, H311L; 3, H311S; 4, H311A.

Supplementary Figure 2

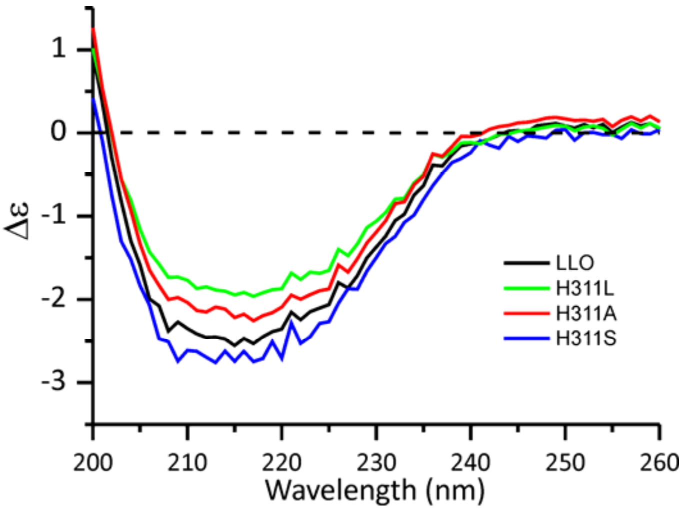

Supplementary Figure 2. Far-UV circular dichroism spectra of LLO and its mutants

Proteins were diluted in 20 mM MES, 10 mM NaCl, pH 5.7 at a final concentration of 0.2 mg/ml. Temperature was 20 °C.

Supplementary Figure 3

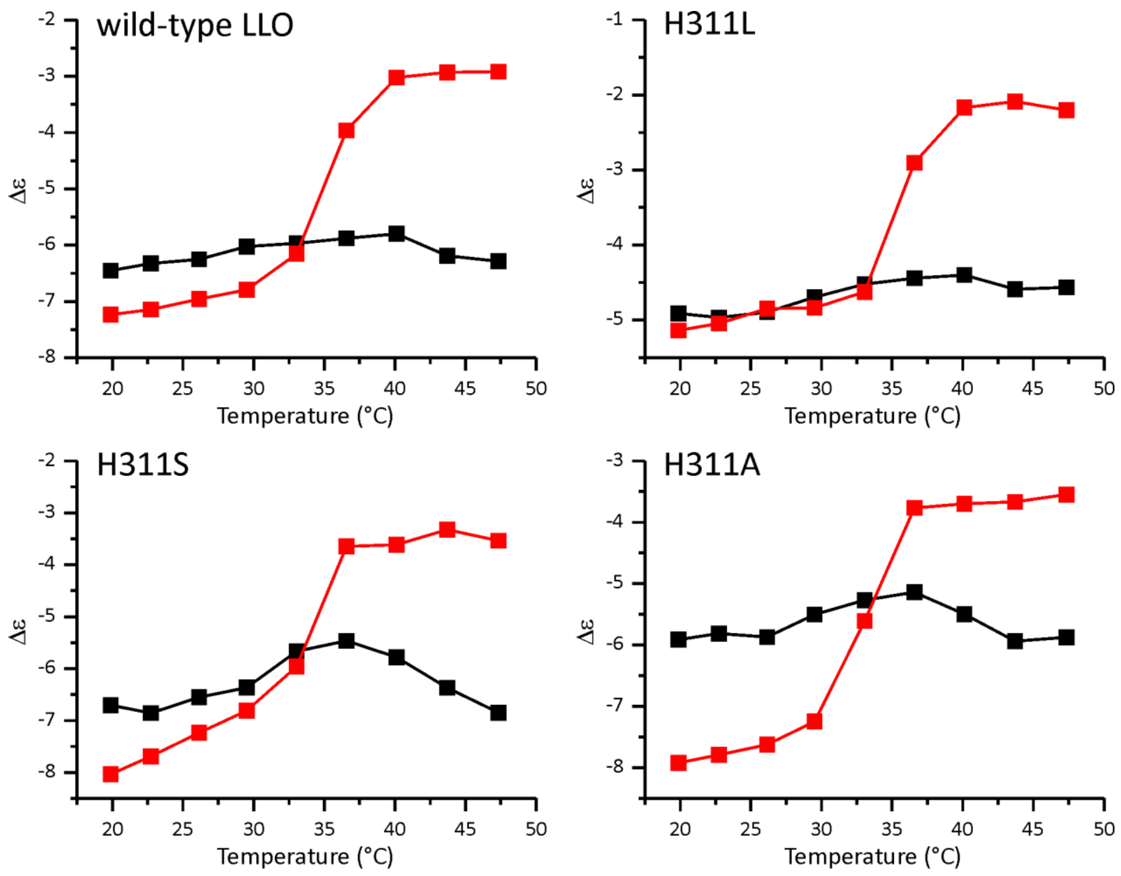

Supplementary Figure 3. Unfolding of proteins as measured by circular dichroism spectroscopy

Spectra were taken at pH 5.7 (black) or 7.5 (red). Protein concentration was 0.2 mg/ml.

Supplementary Figure 4

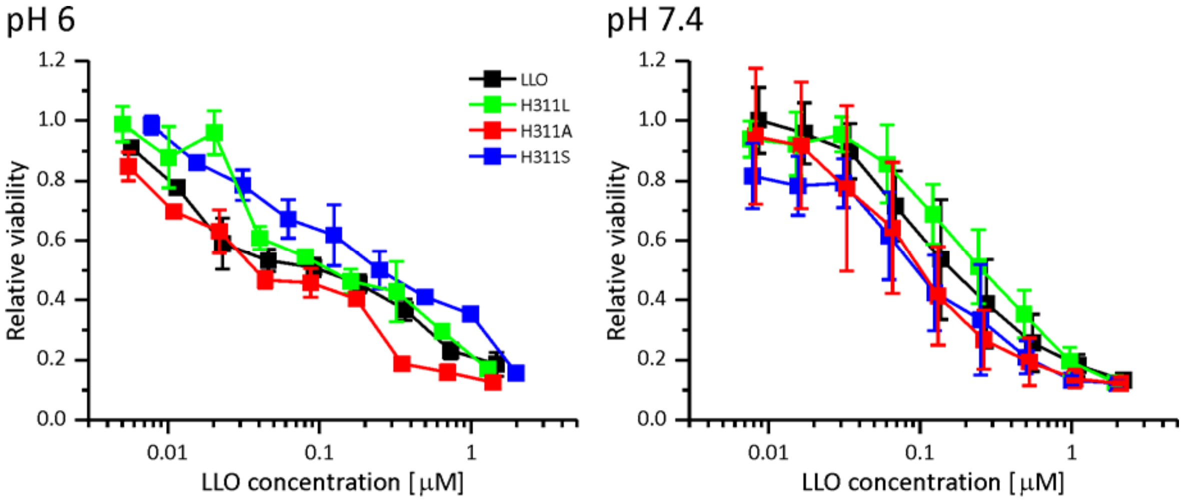

Supplementary Figure 4. Toxicity of proteins used in the study towards Caco-2 cells

Proteins were added to Caco-2 cells at respective pH, incubated for 3 h at 37 °C, washed with fresh medium, incubated overnight and then assayed with MTT reagent. The averages ± S.D. of three to four parallels of two different independent experiments are presented.
